# Rethinking navel size thresholds for omphalitis diagnosis in newborn dairy calves using machine learning

**DOI:** 10.3168/jdsc.2025-0937

**Published:** 2026-02-06

**Authors:** A. Rico, A.F.A. Pires, N. Silva-del-Rio

**Affiliations:** 1 Department of Population Health and Reproduction, UC Davis School of Veterinary Medicine, Davis, CA 95616; 2 UC Davis Veterinary Medicine Teaching and Research Center, Tulare, CA 93274

## Abstract

Omphalitis in newborn calves is an underreported disease with no gold standard diagnostic test. Comprehensive examination is often impractical on farms, and simplified protocols based on the umbilical stump diameter have been proposed. However, existing thresholds relied on expert opinion or limited clinical evidence. The objectives were to (1) assess the reliability of a technique for measuring umbilical stump diameter by evaluating intra- and interobserver agreement; (2) develop a statistical model describing umbilical stump diameter as a function of disease status in Holstein (HO) and Jersey (JE) heifers aged 3 to 10 d; and (3) propose thresholds to estimate farm-level prevalence (optimum sensitivity [Se] and specificity [Sp]) and to identify umbilici with a high probability of omphalitis (maximize Sp). The study enrolled newborn HO and JE heifers from commercial farms in the San Joaquin Valley (California): 157 calves for intraobserver analysis, 44 for interobserver analysis (3 raters), and 667 for threshold estimation. Intra- and interobserver agreement were assessed using the CV and intraclass correlation coefficient (ICC), respectively. Because true disease status was unknown, thresholds were derived using an unsupervised machine-learning approach that inferred healthy and diseased subpopulations from the observed data. Thresholds were estimated separately by breed using 2-component Gaussian mixture models under the biological assumptions that (1) stump diameters arise from a mixture of healthy and diseased subpopulations, (2) diseased umbilici are larger on average, (3) diameters within each subpopulation follow a normal or log-normal distribution, and (4) distributions are stable in calves aged 3 to 10 d across herds. Receiver operating characteristic (ROC) analysis was applied to a simulated sample to estimate diagnostic test performance. Umbilical stump diameter measurements at the external umbilical ring were reliable with good (CV = 5.94% [95% CI: 5.31–6.57]) and fair to good (ICC = 0.76 [95% CI: 0.65–0.85]) agreement for intra- and interobserver agreement, respectively. The breed-specific models accurately described the observed data. Diagnostic tests based on umbilical stump diameter can discriminate between healthy and diseased umbilici (HO area under the curve [AUC] of 0.56 [95% CI: 0.51–0.62]; JE AUC of 0.65 [95% CI: 0.58–0.72]). Thresholds optimizing the Se and Sp trade-off (HO: 16.5 mm; JE: 13.0 mm) achieved high Sp (0.91 and 0.97) but low Se (0.35 and 0.46) for HO and JE. Higher thresholds prioritizing Sp (HO: 19.3 mm; JE: 14.9 mm) yielded Sp ≥0.99, with Se of 0.22 and 0.28. The identified threshold should be interpreted cautiously given the low Se and modest ROC values. Further validation in independent populations and associations with clinical findings are needed.

## Highlights

• Measuring the umbilical stump diameter is a precise and reliable tool.
• Machine learning offers an alternative in the absence of a gold standard.
• Umbilical stump diameter differentiates between healthy and diseased umbilici.

## Graphical Abstract

**Figure:**
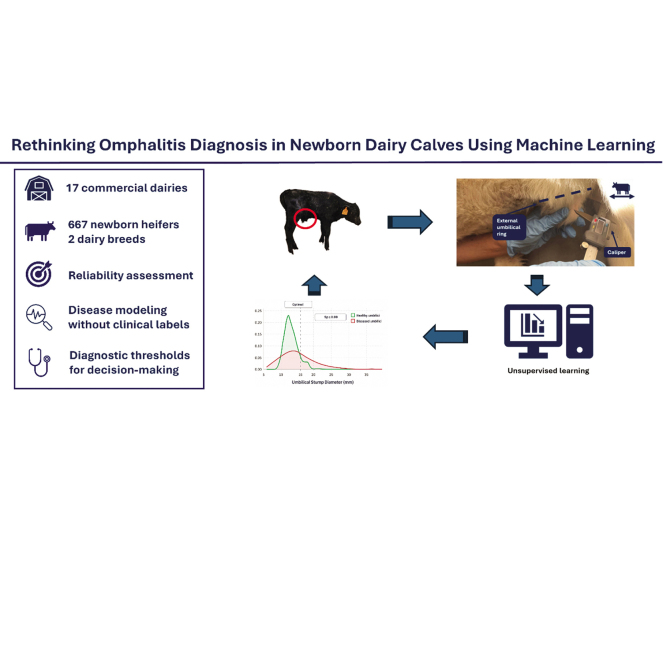
**Summary:** Omphalitis in newborn calves is an overlooked disease with no gold-standard diagnostic test. Umbilical stump diameter has been proposed as a proxy for clinical status, but existing thresholds relied on expert opinion, and their clinical performance is unknown. Using machine learning leveraged by veterinary expertise and observed samples from 437 Holstein and 230 Jersey newborn heifers across 17 dairy farms in California (US), our study proposed new thresholds to diagnose omphalitis. This methodology allows for estimating the clinical performance of the diagnostic test without the need to determine the clinical condition of the study subjects. We propose 2 thresholds: one to estimate farm-level prevalence (16.5 mm for Holstein, 13.0 mm for Jersey) and another to identify calves likely to be diseased (19.3 mm for Holstein, 14.9 mm for Jersey).

During gestation, the bovine fetus is connected to the placenta by the umbilical cord, which contains 2 arteries, 1 vein, and the urachus. After parturition and cord rupture, all umbilical structures persist transiently as remnants, with a portion forming an external umbilical stump. In the early neonatal period, these highly vascular tissues may remain moist, creating favorable conditions for bacterial colonization (Stewart et al., 2016). Infection of umbilical remnants is termed omphalitis, also known as navel disease.

In healthy calves, the umbilical remnants involute rapidly within the first 24 to 48 h of life (Robinson et al., 2015; von Konigslow et al., 2022). Bacterial contamination may cause omphalitis, delaying involution. Clinically omphalitis is characterized by peri-umbilical swelling, local heat, pain on palpation, and variable purulent discharge. Extension of infection along specific umbilical remnants may result in cystitis or pyuria, intra-abdominal abscessation, or septic phlebitis and hepatic abscessation, with possible progression to septicemia, septic arthritis, peritonitis, or death (Virtala et al., 1996; House et al., 2020).

Although omphalitis can affect health, it is frequently underdiagnosed. Producer-reported prevalence has been as low as 2% (USDA, 2018), whereas in-depth assessments have identified prevalence closer to 15% to 30% (Wieland et al., 2017; Dachrodt et al., 2021). This discrepancy indicates that mild infections may go unnoticed during routine management.

Beyond acute illness, umbilical infections impair productivity and welfare. Omphalitis reduces ADG during the preweaning period, and growth declines further with longer infections (Virtala et al., 1996). Early-life diseases, including umbilical infections, may also compromise heifer development and long-term performance (Abuelo et al., 2021).

Identifying omphalitis under commercial field conditions is challenging because no validated gold standard exists for diagnosis in live calves, and confirmation through necropsy is ethically problematic because it would require euthanizing otherwise viable newborn animals (FASS, 2020). In addition, no standardized field protocol exists for classifying umbilical disease (Van Camp, 2021). Physical examination of the umbilicus typically includes evaluation of stump size, discharge, pain response, and the presence of fistulas (House et al., 2020); however, comprehensive examinations are often impractical on commercial dairies due to time and labor constraints. Ultrasonography supports assessment of extra-abdominal and intra-abdominal umbilical structures, but requires specialized equipment, trained personnel, and additional handling time, limiting its large-scale use. Moreover, ultrasonography does not appear more sensitive than physical examination during the first days of life, when disease onset is most common (Wieland et al., 2017). Consequently, simplified protocols based on specific clinical signs, such as swelling, abscesses, or discharge, have been proposed to screen calves (Van Camp, 2021).

In research settings, the diameter of the umbilical stump has been used as indicator of disease (Grover and Godden, 2010; Wieland et al., 2017; Steerforth and Van Winden, 2018). Proposed thresholds include 25 mm (Wieland et al., 2017), 13 mm (Steerforth and Van Winden, 2018), and 12.7 to 15.9 mm (Grover and Godden, 2010). These values have been primarily based on expert opinion, and their sensitivity (**Se**) and specificity (**Sp**) remain unknown. Ideally, diagnostic thresholds should rely on clinical performance. However, this requires knowing the true disease status (Zweig and Campbell, 1993), which is particularly challenging for omphalitis because no validated diagnostic gold standard exists. As an alternative, unsupervised machine learning–based clustering can identify hidden clinical patterns (healthy and diseased subpopulations) from observed measurements, such as umbilical stump diameter, by relying on biologically informed assumptions rather than predefined disease-labeled data (Nahm, 2022; Trapanese et al., 2024).

In this observational study, the objectives were to (1) assess the reliability of a technique for measuring umbilical stump diameter by evaluating intra- and interobserver agreement, (2) develop a statistical model describing umbilical stump diameter as a function of disease status in Holstein (**HO**) and Jersey (**JE**) calves aged 3 to 10 d, and (3) propose thresholds to estimate farm-level prevalence and identify umbilici with a high probability of omphalitis.

Umbilical stump diameter was measured using a digital caliper, as described by von Konigslow et al. (2022). Briefly, navel measurements were taken as close as possible to the external umbilical ring. To ensure consistency, the observer stabilized the stump by clamping the surrounding skinfold with one hand. The umbilical stump diameter was measured in the latero-lateral direction by gently applying the caliper jaws against the stump, avoiding tissue compression while ensuring slight pressure to prevent movement.

For the reliability assessment, a convenience sample of 4-d-old heifers from 2 commercial raising operations in California was enrolled. Intraobserver agreement was evaluated in November 2022, when a single rater (AR) performed 3 repeated measurements on 157 calves. Interobserver agreement was assessed in July 2023, involving 3 veterinary residents who each measured the same 44 calves once. All raters were trained by AR through an in-person demonstration and supervised practice on 10 calves. In addition, AR received training following von Konigslow et al. (2022), supplemented by on-farm practice and consultation with the author.

For the threshold estimation, the study population consisted of a convenience sample of 667 female calves, including 437 HO and 230 JE, between 3 and 10 d old, originally collected for other research purposes (i.e., as part of a cross-sectional study). These calves were enrolled from 17 source dairies in the San Joaquin Valley (California), between September and December 2022. Participating farms were identified through the University of California Agriculture and Natural Resources Extension, dairy consultants, and veterinarians, and were selected based on willingness to participate. Calves from 10 of the 17 dairies were raised on site. One of these farms also raised calves from 2 additional dairies under the same ownership, all within 15 km of each other. Calves from the other 5 dairies were raised at a single commercial facility that received animals from distances between 10 and 85 km. For each calf, the umbilical stump diameter was measured once by AR or a trained technician using the previously described technique. Additional data collected included calf identification, source dairy, age, and breed.

Statistical analysis was performed using R (version 4.3.2; https://www.r-project.org). Statistical significance was declared at 0.05.

Study populations were described using means and SD for continuous variables and proportions for categorical variables. Normality was assessed using the Shapiro–Wilk test, and data were either transformed or analyzed with the nonparametric methods when appropriate.

Intraobserver agreement was assessed using the CV, calculated as the standard deviation divided by the mean and expressed as a percentage. The CV was first computed for each set of triplicate measurements within the same calf and then averaged across all calves (Szklo and Nieto, 2019). Interobserver agreement was assessed using the intraclass correlation coefficient (**ICC**), calculated with a 2-way mixed-effects model using the R package *psych* (version 2.4.1; McGraw and Wong, 1996; Szklo and Nieto, 2019; Revelle, 2024). Both estimates are reported with their 95% CI to convey the uncertainty of the results. Agreement between the 2 indices was also evaluated visually using Bland–Altman plots.

The statistical model describing umbilical stump diameter as a function of disease status was based on the hypothesis that the observed sample of stump diameters represents a mixture of 2 underlying populations: calves with healthy umbilici and calves with diseased umbilici. The following biological assumptions were made: (1) umbilical stump diameters of healthy and diseased calves follow 2 distinct normal or log-normal distributions (Tan et al., 2022); (2) diseased umbilici are, on average, larger than healthy umbilici (Grover and Godden, 2010; Wieland et al., 2017; Steerforth and Van Winden, 2018); and (3) the definition of healthy and diseased umbilici remains unchanged for newborn calves aged between 3 and 10 d and across farms (Robinson et al., 2015; von Konigslow et al., 2022).

Observed data were Box–Cox transformed, and the parameters of each distribution (i.e., healthy and diseased umbilici) were subsequently estimated using finite mixture models and the expectation-maximization algorithm configured to fit 2 normal components (Aiemjoy et al., 2020; Tan et al., 2022). The analysis was conducted separately for each breed, as their observed umbilical stump diameters differed significantly (Wilcoxon rank-sum test; *P* < 0.001), using the R package *mixtools* (version 2.0.0; Benaglia et al., 2009). Consequently, 4 distributions were characterized, defined by the mean, SD, and weight of each component within a breed: HO calves with healthy umbilici, HO calves with diseased umbilici, JE calves with healthy umbilici, and JE calves with diseased umbilici.

Following model fitting, 2 subsets per breed of simulated data were generated through parametric simulation using the corresponding estimated parameters and observed sample size. Within each breed, its 2 subsets were combined to form a simulated sample equal in size to breed-specific observed sample. In summary, this procedure produced a replication of the observed data for each breed. Finally, goodness of fit was assessed by comparing the observed samples, in which clinical status was unknown, with the simulated samples, in which clinical status was known, using the Wilcoxon rank-sum test. The parametric simulation and goodness-of-fit assessment were repeated 1,000 times, with the number of iterations chosen arbitrarily.

Breed-specific simulated samples were randomly generated for further analysis after confirming no distributional differences from the observed data using the Wilcoxon rank-sum test. Within each breed, the R package *pROC* v1.18.5; Robin et al., 2011) was used to plot the receiver operating characteristic (**ROC**) curve, calculate the area under the curve (**AUC**), and identify decision thresholds. Because umbilical stump diameter was expected to be a continuum with substantial overlap between healthy and diseased distributions, no single cutoff is perfect. Therefore, 2 thresholds were defined a priori for distinct purposes: one balancing Se and Sp to support herd-level prevalence estimation, based on Youden's J index (Robin et al., 2011), and a more conservative threshold prioritizing Sp to identify calves with a high probability of disease for clinical decision-making (lowest threshold achieving Sp ≥0.99). Bootstrap resampling was used to estimate Se and Sp, with results reported as the median and 5th–95th percentiles.

The intraobserver agreement (n = 157), expressed as a CV of 5.94% (95% CI: [5.31, 6.57]), was classified as very good. The interobserver agreement (n = 44), measured by an ICC of 0.76 (95% CI: [0.65, 0.85]), was classified as fair to good (Szklo and Nieto, 2019). The Bland–Altman plots suggested a good agreement (Figure 1), as the mean differences did not differ statistically from 0: −0.13 mm (95% CI: [−0.83, 0.57]) in the intraobserver trial and −1.26 mm (95% CI: [−3.82, 1.29]) in the interobserver trial. The individual differences between repeated measurements for each calf did not show any systematic pattern in the intraobserver assessment. However, one of the raters in the interobserver trial consistently recorded slightly lower measurements for larger umbilical stump diameters compared with the other 2 raters.

**Figure 1:**
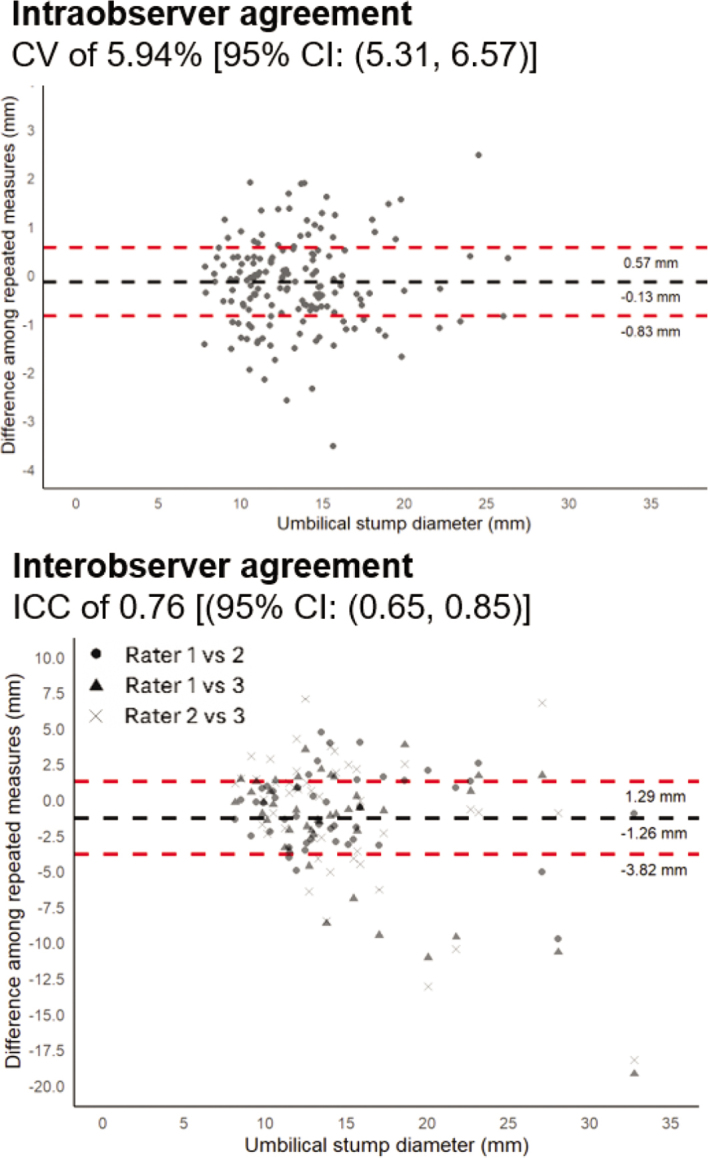
Bland–Altman plots were used to visually assess the reliability of umbilical stump diameter measurements. Intraobserver agreement was evaluated using the CV based on 3 repeated measurements per calf in 157 calves by a single rater; each dot in the plot represents the average difference among the 3 repeated measures for an individual calf. Interobserver agreement was assessed using the intraclass correlation coefficient (ICC) based on measurements from 3 different raters, each measuring the same 44 calves once; each marker represents the difference between 2 raters for the same calf. In both plots, the black dashed line indicates the mean difference, and the outer red dashed lines show the 95% CI. The x-axis displays the average umbilical stump diameter (mm) per calf across repeated measurements.

The observed umbilical stump diameter was significantly larger in HO (n = 437) than in JE (n = 230; *P* < 0.001). The average diameter was 14.9 mm (SD = 4.7) for HO and 12.8 mm (SD = 4.6) for JE, with ranges of 5.6 to 41.4 mm and 5.2 to 35.6 mm, respectively. Additionally, the average age of HO was 5.8 d (SD = 2.1) and 5.4 d (SD = 1.9) in JE, with both groups ranging from 3 to 10 d.

The breed-specific distributional parameters of umbilical stump diameter by disease status are shown in Table 1, with all values presented on the original (back-transformed) scale.

**Table 1:** Unsupervised statistical learning–derived, breed-specific distribution parameters for healthy and diseased umbilici based on umbilical stump diameter, and the expected diagnostic performance of different decision thresholds for identifying omphalitis in newborn dairy calves^1^

Item	Holstein	Jersey
Healthy umbilici subpopulation^2^ (mm)		
Average ± SD	13.7 ± 1.1	10.7 ± 1.0
Middle 95%^3^	10.9–18.9	8.4–13.0
Diseased umbilici subpopulation^2^ (mm)		
Average ± SD	14.4 ± 1.2	12.6 ± 1.1
Middle 95%^3^	7.5–32.9	7.2–28.9
Area under the ROC curve^4^	0.56 (0.51, 0.62)	0.65 (0.58, 0.72)
Optimal threshold^5^ (mm)		
Threshold	16.5	13.0
Se^4^	0.35 (0.29, 0.41)	0.46 (0.38, 0.54)
Sp^4^	0.91 (0.86, 0.94)	0.94 (0.92, 1.00)
Lowest threshold (mm) achieving Sp ≥0.99		
Threshold	19.3	14.9
Se^4^	0.22 (0.16, 0.27)	0.28 (0.21, 0.35)

1 Estimates are based on umbilical stump diameter measured at the external umbilical ring using observed samples from 437 Holstein and 230 Jersey heifers across 17 California dairy farms. ROC = receiver operating characteristic; Se = sensitivity; Sp = specificity.

2 Subpopulations were assumed to follow normal or log-normal distributions. Data were Box–Cox transformed before analysis, and results are presented on the original scale (i.e., back-transformed).

3 The interval between the 2.5th and 97.5th percentiles.

4 95% CI reported in parentheses.

5 Best trade-off between Se and Sp.

For HO calves, the estimated weights of the healthy and diseased subpopulations were 0.49 and 0.51, respectively. The mean umbilical stump diameter was 13.7 mm (SD = 1.1) for the healthy umbilici and 14.4 mm (SD = 1.2) for the diseased umbilici. Based on modeled distributions, 95% of healthy calves are expected to fall within 10.9 to 18.9 mm, whereas diseased calves are expected to fall within 7.5 to 32.9 mm. In 997 out of 1,000 iterations, no significant differences were found between the observed and simulated samples (*P* > 0.05; Figure 2). The ROC analysis showed an AUC of 0.56 (95% CI: [0.51, 0.62]). The optimal estimated threshold, identified at 16.5 mm, provided an estimated Se of 0.35 (95% CI: [0.29, 0.41]) and an estimated Sp of 0.91 (95% CI: [0.86, 0.94]). The lowest threshold achieving Sp ≥0.99 was 19.3 mm, with a Se of 0.22 (95% CI: [0.16, 0.27]; Table 1).

**Figure 2:**
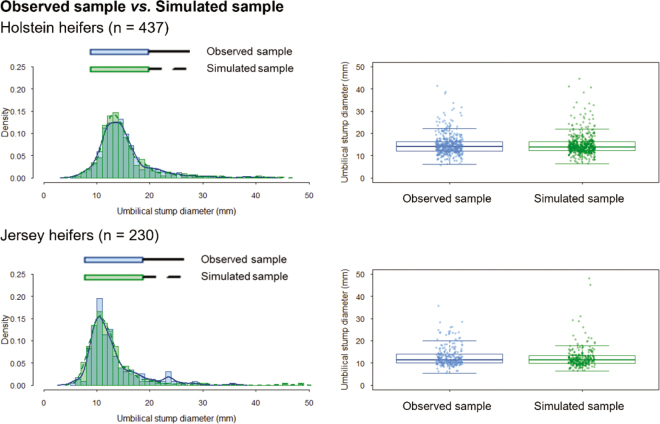
Goodness of fit was visually assessed by comparing observed samples (with unknown clinical status) to simulated samples (with assigned clinical status) using histograms and boxplots. The midline represents the median, the box represents the interquartile range (IQR), and the whiskers extend to 1.5 × IQR. The statistical model, developed through unsupervised learning to describe umbilical stump diameter as a function of disease status, was based on biologically plausible assumptions and supported by the visual agreement between observed and simulated data. In addition, the Wilcoxon rank-sum test was used to statistically compare the distributions, with nonsignificant results (*P* > 0.05) indicating no evidence of difference.

Similarly, for JE calves, the estimated weights of the healthy and diseased subpopulations were 0.39 and 0.61, respectively. The mean umbilical stump diameter was 10.7 mm (SD = 1.0) for the healthy umbilici and 12.6 mm (SD = 1.1) for the diseased umbilici. Based on modeled distributions, 95% of healthy calves are expected to fall within 8.4 to 13.0 mm, whereas diseased calves are expected to fall within 7.2 to 28.9 mm. In 994 out of 1,000 iterations, no significant differences were found between the observed and simulated samples (*P* > 0.05; Figure 2). The ROC analysis showed an AUC of 0.65 (95% CI: [0.58, 0.72]). The optimal threshold, identified at 13.0 mm, provided a Se of 0.46 (95% CI: [0.38, 0.54]) and a Sp of 0.97 (95% CI: [0.92, 1.00]). The lowest threshold achieving Sp ≥0.99 was 14.9 mm, with a Se of 0.28 (95% CI: [0.21, 0.35]; Table 1).

This study supports the umbilical stump diameter is a reliable measurement. We developed a statistical model describing stump diameter as a function of disease status, enhancing the biological interpretation of numerical measurements. By modeling the observed distribution from commercial farms as a mixture of 2 subpopulations and applying unsupervised clustering techniques leveraged by biological assumptions, we established decision thresholds without requiring a clinical gold standard. These thresholds provide a practical tool for identifying omphalitis cases and serve as benchmarks for herd-level monitoring. Effective herd health decisions rely on measurable indicators. Without at least an estimate of prevalence, it is difficult to plan studies, interpret data, or implement evidence-based strategies (Rogan and Gladen, 1978).

The levels of intraobserver and interobserver agreement in our study were consistent with those reported by von Konigslow et al. (2022), with ICC of 0.73 (95% CI: 0.42–0.92) and 0.89 (95% CI: 0.69–0.97), respectively. Intraobserver agreement was similar across the observed range of umbilical stump diameters, suggesting consistent measurements regardless of stump size (Figure 1). Interobserver variability was slightly higher for larger umbilici, which may reflect the greater difficulty of obtaining consistent measurements in stumps affected by inflammation or calf discomfort (Figure 1). Despite this variability, differences between the raters were not considered substantial enough to compromise classification. In our study, the raters were trained before navel evaluation. Although the results reflect the reliability of the specific raters involved (Koo and Li, 2016), training and standardization help to ensure reliable measurements. All 5 observers were considered comparable in terms of graduate level education, with 4 being veterinary clinicians with years of dairy practice experience.

The study population consisted of 2 dairy breeds. Umbilical stump diameter was significantly larger in HO compared with JE. Von Konigslow et al. (2022) reported a positive association between umbilical stump diameter and BW, with larger calves having greater diameters. This supports our decision to analyze HO and JE heifers as distinct populations, given the substantial difference in body size between breeds. Ignoring this distinction could result in underestimation or overestimation of navel disease within each breed.

Several stump diameter thresholds had already been proposed (Grover and Godden, 2010; Wieland et al., 2017; Steerforth and Van Winden, 2018). However, because their Se and Sp were unknown, they may be overly conservative and risk misclassification. The clinical performance of a diagnostic test is essential for establishing decision thresholds with practical value. The ROC analysis is a widely used method to assess diagnostic accuracy, but it requires that the clinical condition of the study subjects be known (Zweig and Campbell, 1993). Determining clinical status is often challenging, especially in absence of gold standard, and time-consuming, as it depends on trained personnel, manual evaluation, and high-quality disease data (Zweig and Campbell, 1993; Trapanese et al., 2024). In this context, unsupervised statistical learning techniques, such as the algorithm applied in this study, can address these challenges by identifying hidden patterns associated with clinical conditions (Trapanese et al., 2024), relying on biologically plausible assumptions.

Our models suggest that a diagnostic test based on umbilical stump diameter can discriminate between diseased and healthy umbilici in dairy heifers aged 3 to 10 d, as the AUC was significantly greater than 0.5 for both breeds. The optimal thresholds, 16.5 mm for HO and 13.0 mm for JE, offered high Sp but only fair to poor Se, correctly identifying healthy umbilici while missing a proportion of diseased ones. This was expected, as previous research showed similar stump diameters in calves with and without clinical signs (Grover and Godden, 2010). Interestingly, our model also linked very small diameters with disease. Although counterintuitive, this aligns with human neonatology studies, where abnormally small stump size may reflect underlying health issues rather than optimal healing (Proctor et al., 2013; Lee et al., 2020). While optimal thresholds are useful for estimating herd-level prevalence due to their balanced Se and Sp (Rogan and Gladen, 1978), thresholds that maximize Sp are better suited for guiding clinical interventions (i.e., antibiotic therapy), as the probability of healthy calves having larger umbilical stump diameters is very low. Based on our model, the lowest thresholds achieving Sp ≥0.99 were 19.3 mm for HO and 14.9 mm for JE.

The diagnostic performance of these tests should be interpreted with caution. The modest AUC and limited Se indicate that stump diameter is best viewed as a screening measure rather than a definitive diagnostic test. Accordingly, the proposed thresholds serve as decision-making aids: to support herd-level monitoring and prevalence estimation, and to identify calves with a high probability of disease when considering clinical intervention.

The limitations of this study arise in the dependence on model-based assumptions inherent to unsupervised learning, and the absence of a universal method for validating the results. To partially assess the model's performance, we relied on goodness-of-fit diagnostics, which showed no statistically significant differences between the transformed observed data and the simulated samples (Figure 2). The thresholds were also derived exclusively from female calves in a convenience sample of farms, which may limit external validity. A strength of this study, however, is the inclusion of calves from 17 different herds, rather than from a single source, which adds population variability and reduces the influence of herd-specific patterns. Another strength is that both subpopulations (healthy and diseased umbilici) were represented under field conditions, without artificial modification. Further validation in independent populations and evaluation of associations with detailed clinical findings are warranted.
